# 

**Published:** 1888-12

**Authors:** 


DECEMBER, 1888.

### CONTENTS.

ORIGINAL PAPER:— *Page*

Some Recent Developments of the Germ Theory, more particularly in Relation to the Treatment of Phthisis. By R. SHINGLETON SMITH, M.D. Lond., B.Sc., F.R.C.P. ... ... ... ... ... ... ... ... 225

CLINICAL RECORDS:—

Cases Illustrating the Surgery of the Thyroid Body. By W. H. HARSANT, F.R.C.S. ... ... ... ... ... ... ... ... ... ... ... ... 265

A Case of Raynaud's Disease. By HENRY WALDO, M.D., M.R.C.P. ... 272

REVIEWS OF BOOKS ... ... ... ... ... ... ... ... ... ... ... ... ... ... 276

NOTES ON PREPARATIONS FOR THE SICK ... ... ... ... ... ... ... ... 291

PERISCOPE ... ... ... ... ... ... ... ... ... ... ... ... ... ... ... 293

SCRAPS ... ... ... ... ... ... ... ... ... ... ... ... ... ... ... ... 299

